# Rhein attenuates obesity-related glomerulopathy by inhibiting the P2X7R/NLRP3 inflammasome pathway and protecting podocytes

**DOI:** 10.22038/ijbms.2025.88054.19020

**Published:** 2025

**Authors:** Lifang Wei, Jinwen Zhang, Liangding Dou, Xiaoxin Wu, Minmin Xu, Jinxia Ye, Yanyan Yang, Yongxing Zhang, Shaojian Xiao

**Affiliations:** 1 Department of Nephrology, The Third People’s Hospital Affiliated to Fujian Univer-sity of Traditional Chinese Medicine, Fuzhou, Fujian, China; 2 State Key Laboratory of Infectious Disease Vaccine Development, Xiang An Bio-medicine Laboratory & State Key Laboratory of Molecular Vaccinology and Molecular Diagnostics, School of Public Health, Xiamen University, Xiamen, Fujian, China; 3 State Key Laboratory for Diagnosis and Treatment of Infectious Diseases, National Clinical Research Centre for Infectious Diseases, the First Affiliated Hospital, Zhejiang University School of Medicine, Hangzhou 310003, Zhejiang, China; 4 Academy of Integrative Medicine, Fujian University of Traditional Chinese Medicine, Fujian Key Laboratory of Integrative Medicine on Geriatrics, Fuzhou, Fujian, China; 5 Innovation and Transformation Center, Fujian University of Traditional Chinese Medicine, Fuzhou, Fujian, China; 6 The Third People’s Hospital Affiliated to Fujian University of Traditional Chinese Medicine, Fuzhou, Fujian, China

**Keywords:** Inflammation, Leptin, NLRP3 Inflammasome, Obesity-related- glomerulopathy, P2X7 receptor, Podocyte, Rhein

## Abstract

**Objective(s):**

To investigate the renoprotective effects of Rhein in obesity-related glomerulopathy (ORG) by inhibiting the P2X7 receptor (P2X7R)/NOD-like receptor protein 3 (NLRP3) inflammasome pathway.

**Materials and Methods:**

ORG was induced in C57BL/6J mice with a high-fat diet (HFD) for 10 weeks, fol-lowed by oral Rhein treatment (70 or 300 mg/kg/day) for 10 weeks. Renal function, histology, and podocyte injury were assessed. *In vitro*, leptin-induced podocyte injury was treated with Rhein or P2X7R antagonists (KN-62 or A-438079). P2X7R/NLRP3 activation, inflammation, and oxidative stress were evaluated.

**Results:**

HFD-induced weight gain, dyslipidemia, renal dysfunction, glomerular hypertrophy, and podocyte injury. Rhein reduced serum triglycerides (TG) and total cholesterol (TC), lowered blood urea nitrogen (BUN), improved urinary protein excretion, and alleviated histological damage. Rhein inhibited P2X7R and NLRP3 activation, down-regulated caspase-1, interleukin (IL)-1β, and IL-18, and restored podocyte markers (Nephrin, Podocin). *In vitro*, Rhein mitigated leptin-induced podocyte injury and inflammasome activation.

**Conclusion:**

Rhein protects against ORG by suppressing the P2X7R/NLRP3 pathway, reducing inflammation and oxidative stress, and preserving podocyte integrity, highlighting its therapeutic potential.

## Introduction

Obesity is a major global public health challenge, with its increasing prevalence leading to various metabolic diseases, including chronic kidney disease (CKD). Among obesity-related renal disorders, obesity-related glomerulopathy (ORG) is characterized by glomerular hypertrophy, podocyte injury, and progressive proteinuria, ultimately resulting in renal dysfunction (1). The pathogenesis of ORG is multifaceted, involving hemodynamic alterations, activation of the renin-angiotensin-aldosterone system (RAAS), insulin resistance, adipokine dysregulation, and chronic inflammation (2, 3). These factors contribute to glomerular hypertension, podocyte stress, and renal inflammation, thereby accelerating disease progression (4, 5).

Podocytes, specialized glomerular epithelial cells essential for the filtration barrier, play a central role in ORG pathogenesis. Podocyte damage disrupts the barrier, causing proteinuria and progressive renal dysfunction (6-8). Leptin, an adipokine elevated in obesity due to resistance, promotes podocyte hypertrophy, proliferation, and fibrosis, thereby exacerbating injury (9, 10).

The NLRP3 inflammasome drives renal inflammation in ORG. The P2X7R, an ATP-gated cation channel, regulates NLRP3 activation upstream by inducing potassium efflux, calcium influx, and mitochondrial dysfunction, which leads to caspase-1 activation and the release of proinflammatory cytokines IL-1β and IL-18 (11-14). Overactivation of this pathway contributes to podocyte dysfunction, inflammation, and fibrosis in ORG (15). Targeting P2X7R/NLRP3 has potential for metabolic kidney diseases, but P2X7R antagonists face limitations in selectivity and efficacy (16, 17).

Rhein, an anthraquinone derived from rhubarb, exhibits anti-inflammatory, anti-fibrotic, and metabolic effects (18-21). It inhibits NLRP3 activation and oxidative stress in conditions such as colitis and arthritis (22-24), but its role in ORG and P2X7R/NLRP3 regulation remains unclear. This study examined Rhein’s renoprotective effects in high-fat diet (HFD)-induced ORG mice and leptin-stimulated podocytes, focusing on P2X7R/NLRP3 inhibition to provide insights for obesity-related kidney diseases.

## Materials and Methods

Animal experiments were approved by the Ethics Committee of Fujian University of Traditional Chinese Medicine (No. FJTCM IACUC2022048). Fifty healthy 6-week-old male C57BL/6J mice were obtained from the Fujian University of Traditional Chinese Medicine Experimental Animal Center. After one week of acclimatization, mice were randomly assigned to two groups: Control (n=10, regular diet) and HFD (n=40, 60% high-fat diet). After 10 weeks, obesity-prone mice (body weight gain >80% of initial) were divided into: HFD (n=8), HFD+Low-dose Rhein (70 mg/kg/day, n=8), and HFD+High-dose Rhein (300 mg/kg/day, n=8) for 10 weeks via oral gavage. At the endpoint, mice were anesthetized, and blood, urine, and kidneys were collected (21).

### Biochemical analysis

The concentration of urinary microalbumin (mALB) was measured by ELISA (Nanjing Jiancheng Bioengineering Institute, China). Serum creatinine (Scr), BUN, TC, low-density lipoprotein (LDL), and TG were analyzed using an automated biochemical analyzer (Nanjing Jiancheng Bioengineering Institute). 24-hour urinary albumin excretion and urinary albumin-to-creatinine ratio (UACR) were determined with standard kits.

### Histological and immunohistochemical analysis

Freshly isolated kidney tissues were fixed in 4% paraformaldehyde, paraffin-embedded, and sectioned (3 μm). Hematoxylin-eosin (HE) staining was used to assess glomerular morphology, and the cross-sectional area was quantified using ImageJ. For immunohistochemistry, sections underwent deparaffinization, rehydration, antigen retrieval, and development using DAB. Antibodies: P2X7R (Proteintech, Wuhan, China) and NLRP3 (Affinity, Jiangsu, China). Sections were visualized by optical microscopy.

### Cell culture and treatment

Conditionally immortalized mouse podocytes (MPC-5; Shanghai Fuheng Biological Company) were cultured in DMEM with 10% FBS at 37 ^°^C and 5% CO₂. Groups: control (medium), leptin (250 ng/ml; Novoprotein, Suzhou, China), leptin+P2X7R antagonists (A-438079, 10 μmol/l or KN-62, 2.5 μmol/l), or leptin+Rhein (10 μg/ml).

### Quantitative real-time PCR (qRT-PCR)

Total RNA was extracted with TRIzol (Beyotime, Shanghai, China), reverse-transcribed (ABM, Vancouver, Canada), and amplified with SYBR Green Master Mix. Primers are in Table 1.

### Reactive oxygen species (ROS) determination

Cells were incubated with DCFH-DA probe (Beyotime); ROS levels were detected using flow cytometry.

### Western blot analysis

Total protein was extracted from renal cortical tissue and podocyte cultures using RIPA lysis buffer. Proteins were extracted with RIPA buffer, separated by SDS-PAGE, transferred to PVDF membranes (LABSELECT, Beijing, China), blocked with 5% skim milk (1 hr), incubated with primary antibodies overnight at 4 ^°^C, then secondary antibodies (1 hr, room temperature), and detected by enhanced chemiluminescence. Bands were quantified with ImageJ.

### Statistical analysis

Data were analyzed with GraphPad Prism. Normality was assessed by the Shapiro-Wilk test. Two-group comparisons were analyzed using Student’s t-test, while multiple groups were analyzed using one-way ANOVA followed by Tukey’s *post-hoc* test. Data are mean±SD; *P*<0.05 was significant.

## Results

### Rhein mitigated HFD-induced weight gain and dyslipidemia

**Figure 1:**
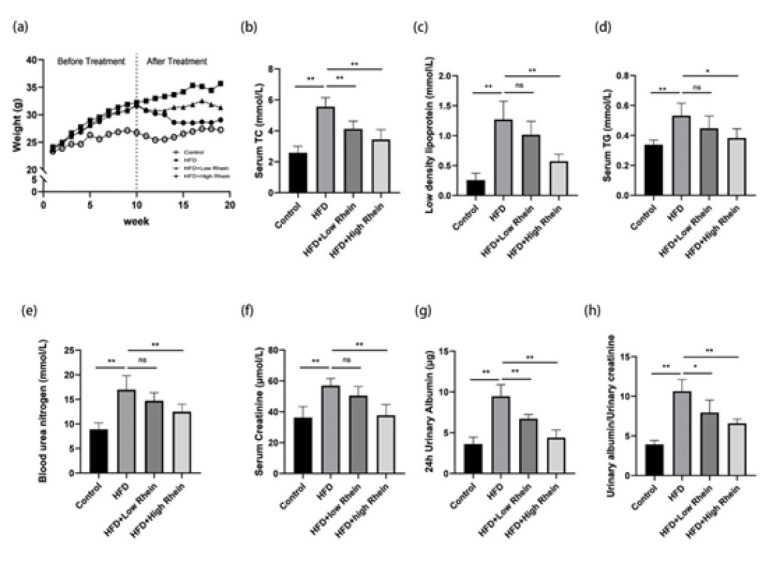
Rhein attenuates obesity-induced renal dysfunction and dyslipidemia in HFD-fed mice

HFD significantly increased body weight compared to controls, but high-dose Rhein attenuated this gain (Figure 1a). HFD elevated serum TC, LDL, and TG (*P*<0.01; Figure 1b-d). High-dose Rhein reduced these levels markedly, with low-dose showing modest effects.

### Rhein attenuated HFD-induced renal dysfunction

HFD raised BUN, Scr, 24-hour urinary albumin, and UACR (*P*<0.01; Figure 1e-h), indicating impairment. High-dose Rhein lowered BUN and Scr; both doses reduced albuminuria and UACR (*P*<0.01).

### Rhein ameliorated HFD-induced glomerular hypertrophy and podocyte injury

**Figure 2:**
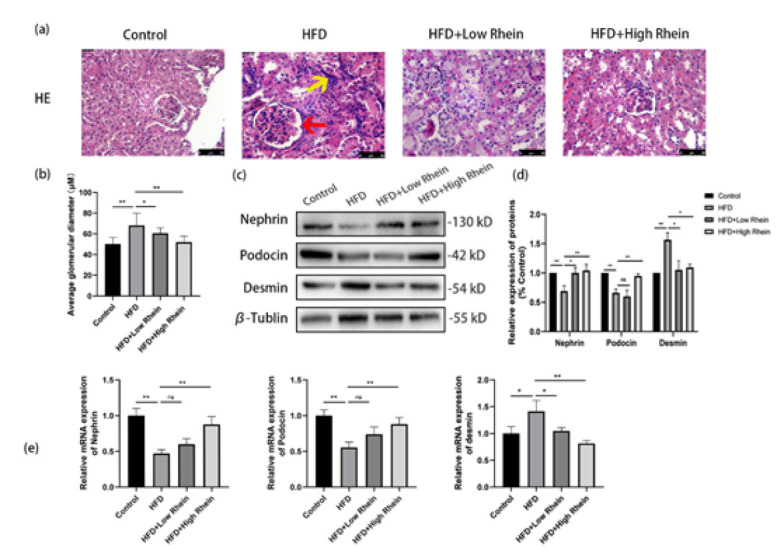
Rhein ameliorates glomerular hypertrophy and podocyte injury in HFD-induced ORG mice

HE staining showed glomerular hypertrophy, inflammatory infiltration (yellow arrows), and mesangial expansion (red arrows) in HFD mice (Figure 2a). Rhein reduced these changes, with high-dose more effective. Glomerular area increased in HFD (*P*<0.01) but decreased with Rhein (*P*<0.05 or *P*<0.01; Figure 2b). Western blots revealed decreased nephrin and podocin, and increased desmin in HFD (*P*<0.01; Figure 2c, d). Rhein restored these markers, with high-dose superior. qRT-PCR confirmed consistent mRNA trends (Figure 2e).

### Rhein suppressed HFD-induced P2X7R/NLRP3 inflammasome activation

**Figure 3:**
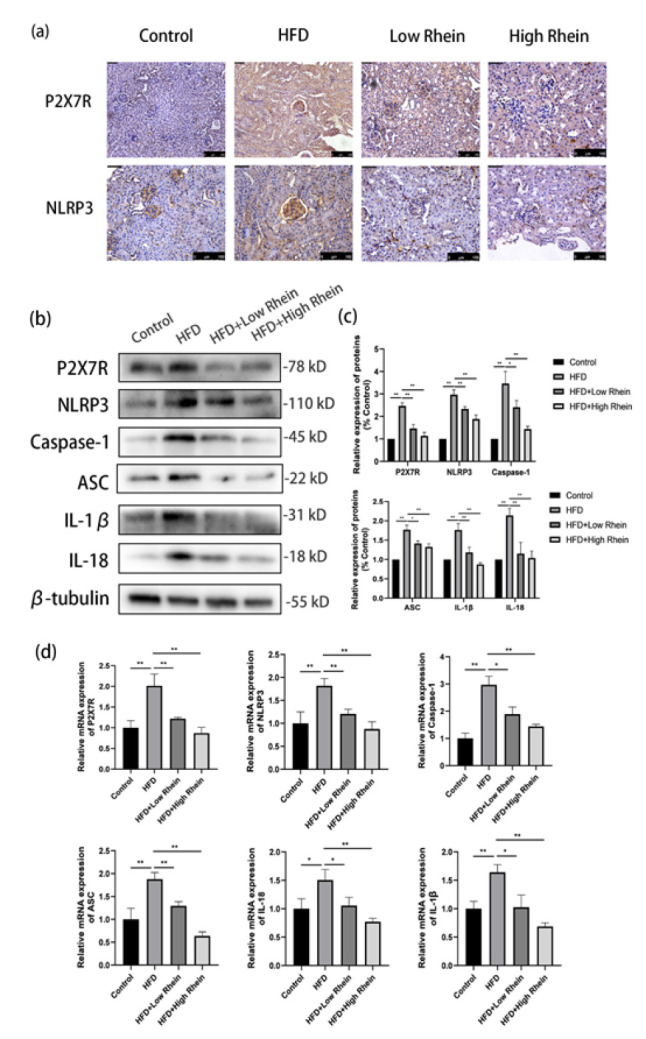
Rhein suppresses P2X7R/NLRP3 activation in HFD-induced ORG mice

Immunohistochemistry revealed elevated P2X7R and NLRP3 expression in HFD kidneys, which was attenuated by Rhein (Figure 3a). Western blots confirmed up-regulated P2X7R, NLRP3, caspase-1, ASC, IL-1β, and IL-18 in HFD (*P*<0.01; Figure 3b, c). qRT-PCR showed similar mRNA increases (*P*<0.001), reduced by Rhein (Figure 3d).

### Rhein alleviated leptin-induced podocyte injury via P2X7R/NLRP3 modulation

**Figure 4:**
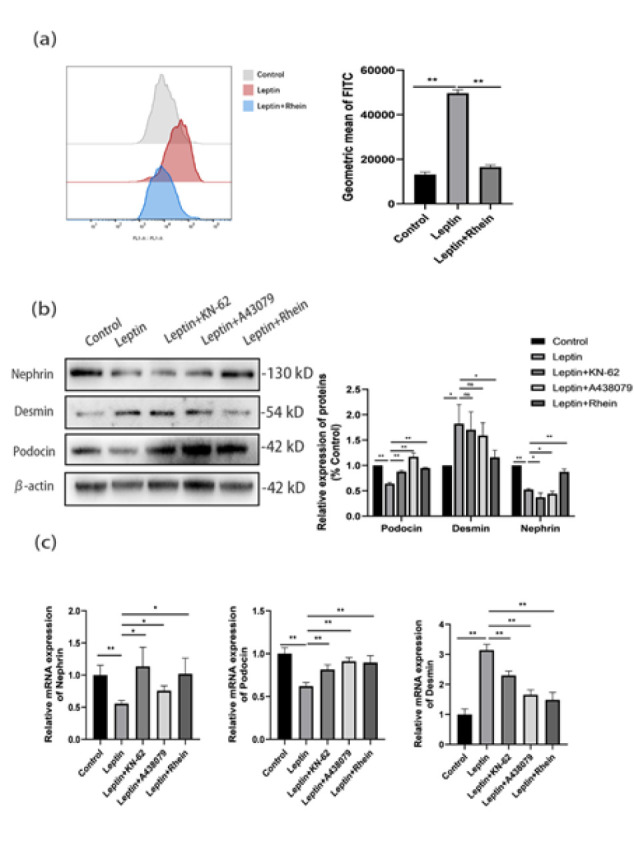
Rhein protects mouse podocytes from leptin-induced injury via the P2X7R/NLRP3 pathway

Leptin increased ROS (Flow-cytometry; *P*<0.01), which was reduced by Rhein (Figure 4a). Western blots showed decreased nephrin/podocin and increased desmin with leptin (*P*<0.01; Figure 4b); however, Rhein and antagonists reversed this effect. qRT-PCR confirmed (Figure 4c).

### Rhein inhibited leptin-induced P2X7R/NLRP3 activation in podocytes

**Figure 5:**
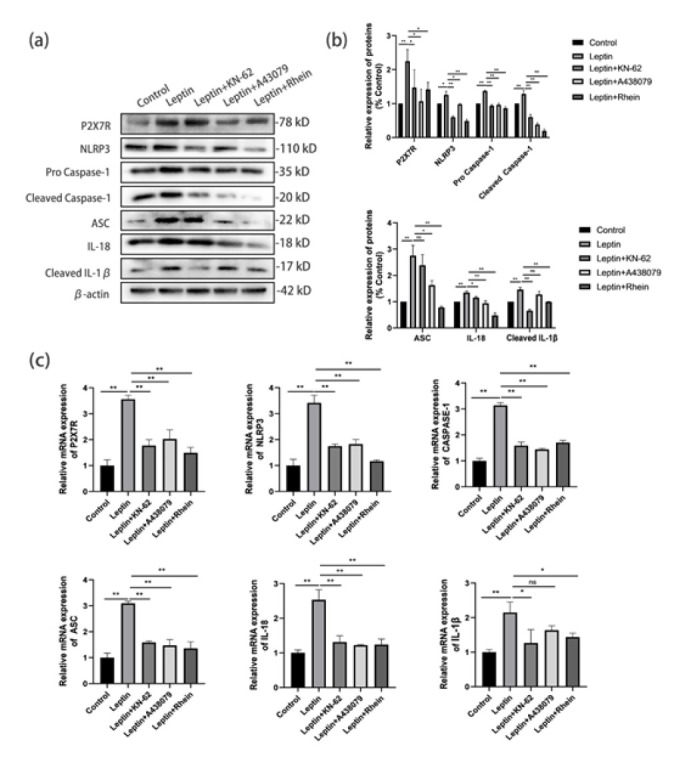
Rhein inhibits leptin-induced P2X7R/NLRP3 activation in mouse podocytes

Leptin up-regulated P2X7R, NLRP3, caspase-1 (pro/cleaved), ASC, IL-18, and cleaved IL-1β (*P*<0.01; Figure 5a, b). Rhein and its antagonists suppressed these effects. qRT-PCR showed similar reductions (Figure 5c).

## Discussion

This study demonstrated that Rhein ameliorated renal dysfunction, inflammation, and podocyte injury in HFD-induced ORG by inhibiting the P2X7R/NLRP3 pathway, supporting its therapeutic potential. 

### Mechanistic insights

Rhein reduced weight gain, dyslipidemia, proteinuria, BUN, glomerular hypertrophy, and inflammation, consistent with early ORG features, such as glomerulomegaly (25). It down-regulated P2X7R/NLRP3, suppressing caspase-1, IL-1β, and IL-18, thereby mitigating inflammation and oxidative stress (11-14). Rhein restored nephrin/podocin and suppressed desmin, thereby protecting podocytes, which are critical for preventing progression to end-stage renal disease (ESRD), in ~30% of ORG patients (6-8, 26). 

### Comparison with existing therapies

Unlike P2X7R antagonists limited by selectivity(16, 17), Rhein matched their effects in leptin models while improving lipids and insulin resistance (27). Leptin drives podocyte damage in ORG, and Rhein’s inhibition via P2X7R/NLRP3 offers a novel target (9, 10, 28). Supporting evidence indicates that NLRP3 blockade ameliorates HFD-induced kidney damage (29).

### Broader implications and limitations

Beyond P2X7R/NLRP3, ORG involves RAAS and insulin resistance(5, 30, 31). Rhein’s multi-target effects align with ORG strategies, such as weight loss (32), and may extend to other kidney diseases (33, 34). Doses (70-300 mg/kg/day) were consistent with prior studies (30, 35), but higher doses risk toxicity (36), necessitating pharmacokinetic studies for translation. Limitations include lack of genetic knockouts and human data; future trials should validate efficacy.

**Table 1:** Primer sequences used for quantitative real-time polymerase chain reaction (qRT-PCR) analysis of selected mouse genes

Target	Species	Forward	Reverse
Nephrin	Mice	TAGTGGACGTGGACGAGGTT	GAGGACAAGAAGCCACTCGC
Podocin	Mice	GGATGGCGGCTGAGATTCTG	AAACCACAGTGGCTGGCTTC
Desmin	Mice	CCAAGCAGGAGATGATGGAATA	CATCCTTTAGGTGTCGGATCTC
P2X7R	Mice	CACCGTGCTTACAGGTGCTA	CGGTCTTGGGGAACTCCTTC
NLRP3	Mice	GTGGTGACCCTCTGTGAGGT	TCTTCCTGGAGCGCTTCTAA
Caspase-1	Mice	TGACAAGAAGGCAAAGGCCG	ACCTCGTCCACGTCCACTAC
ASC	Mice	CTTGTCAGGGATGAACTCAAAA	GCCATACGACTCCAGATAGTAGC
IL-1β	Mice	CGCAGCAGCACATCAACAAG	GTGCTCATGTCCTCATCCTG
IL-18	Mice	GTGAACCCCAGACCAGACTG	CCTGGAACACGTTTCTGAAAGA
β-actin	Mice	CATCCGTAAAGACCTCTATGCCAAC	ATGGAGCCACCGATCCACA

(a) Body weight changes over 20 weeks (dashed line: Rhein start). (b) Serum TC. (c) LDL. (d) TG. (e) BUN. (f) Scr. (g) 24-hour urinary albumin. (h) UACR. *P<0.05, **P<0.01, ns=not significant

HFD: High-fat diet; TC: total cholesterol; LDL: Low-density lipoprotein; TG: Triglycerides; BUN: Blood urea nitrogen; Scr: Serum creatinine; UACR: Urinary albumin-to-creatinine ratio

(a) HE staining (200×; yellow arrows: hypertrophy). (b) Glomerular area (μm²). (c) Western blot of nephrin, podocin, and desmin. (d) Densitometry (β-tubulin normalized). (e) qRT-PCR mRNA. *P<0.05, **P<0.01, ns=not significant

HFD: High-fat diet; ORG: Obesity-related glomerulopathy; HE: Hematoxylin-eosin

(a) IHC (200×; yellow arrows: intense staining). (b) Western blot. (c) Densitometry. (d) qRT-PCR. *P<0.05, **P<0.01, ns

HFD: High-fat diet; ORG: Obesity-related glomerulopathy

(a) ROS flow-cytometry. (b) Western blot. (c) mRNA. *P<0.05, **P<0.01, ns

ROS: Reactive Oxygen Species

(a) Western blot. (b) Densitometry (β-actin normalized). (c) qRT-PCR. *P<0.05, **P<0.01, ns

**Figure 6:**
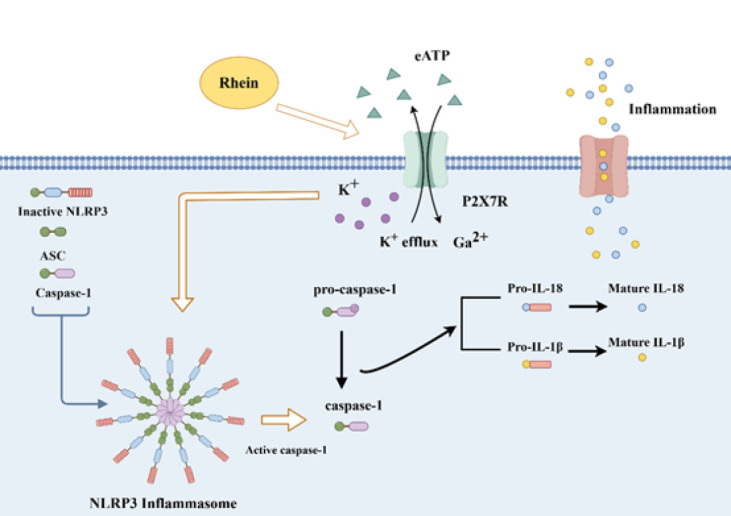
Proposed mechanism of Rhein inhibiting P2X7R/NLRP3 to mitigate inflamma-tion in mice

eATP: Extracellular ATP; P2X7R: P2X7 receptor; ASC: Apoptosis-associated speck-like protein containing a CARD; NLRP3: NOD-, LRR-, and pyrin domain-containing protein 3; IL: Interleukin

## Conclusion

Rhein attenuates ORG by inhibiting P2X7R/NLRP3, reducing inflammation/oxidative stress, and preserving podocytes (Figure 6). 

## Declaration

We have not used any AI tools or technologies to prepare this manuscript.
